# Novel Discoveries Targeting Pathogenic Gut Microbes and New Therapies in Pancreatic Cancer: Does Pathogenic E. coli Infection Cause Pancreatic Cancer Progression Modulated by TUBB/Rho/ROCK Signaling Pathway? A Bioinformatic Analysis

**DOI:** 10.1155/2020/2340124

**Published:** 2020-05-10

**Authors:** Wenhao Luo, Zhe Cao, Jiangdong Qiu, Yueze Liu, Lianfang Zheng, Taiping Zhang

**Affiliations:** ^1^Department of General Surgery, Peking Union Medical College Hospital, Chinese Academy of Medical Sciences and Peking Union Medical College, Beijing 100730, China; ^2^Department of Nuclear Medicine, Peking Union Medical College Hospital, Chinese Academy of Medical Sciences and Peking Union Medical College, Beijing 100730, China; ^3^Clinical Immunology Center, Chinese Academy of Medical Sciences and Peking Union Medical College, Beijing 100730, China

## Abstract

Pancreatic cancer (PC) is a pernicious cancer of the digestive system which remains a high degree of malignancy. Increasing studies demonstrated that regulating the gut microbiome may become a brand new strategy to improve the therapeutic outcomes of PC. This study is aimed at obtaining the pathway in the microbial tumorigenesis of PC. Microarray datasets GSE27890, GSE46234, and GSE17610 were downloaded from the GEO (Gene Expression Omnibus) database. Differential analysis was performed for every single gene chip using the R software package (“Limma” package), and functional enrichment analyses were carried out by DAVID (Database for Annotation, Visualization and Integrated Discovery). The PPI (protein-protein interaction) network was constructed with the Search Tool for the Retrieval of Interacting Genes (STRING). The survival analysis was performed by GEPIA and USCS. A total of 84 differentially expressed genes (DEGs) were identified, and 3 of them were extracted (TUBB, TUBA4A, and TLR5). Biological process analysis revealed that these 3 genes were mainly enriched in pathogenic Escherichia coli (E. coli) infection. Survival analysis and pathway analysis revealed that TUBB (tubulin, beta class I) may be associated with the pathogenic E. coli infection, which may be involved in the carcinogenesis and progression of PC by activating the TUBB/Rho/ROCK signaling pathway. Elevated evidence indicated that a specific gut microbe could affect the progression of PC by suppressing immune response. However, little attention has been paid to the relationship and crosstalk between TUBB/Rho/ROCK signaling, microbes, and PC. This article is aimed at deducing that gut and tumor microbes are related to the development of PC by stimulating TUBB/Rho/ROCK signaling, while ablation of microbes by antibiotics cotreated with inhibitors of TUBB/Rho/ROCK signaling were identified as a novel target for PC therapy.

## 1. Introduction

Pancreatic cancer (PC) is a highly lethal disease with a low overall survival rate. The reason why PC patients have a poor long-term survival rate remains to be explored. Recent studies put up with a novel idea that the pathogenic intestinal bacterium infection may undermine cancer immune surveillance, contributing to chemoresistance, inflammation, and worse patient outcomes [1, 2]. The human intestinal microbes comprise numerous micro-organisms that can influence the host immunity and cancer conditions because gut microbes and the immune system are mutually affected via metabolic crosstalk. A researcher surprisingly discovered that PC is strongly correlated with Gram-negative gammaproteobacteria- (GP-) Escherichia coli (E. coli) [3]. Moreover, Geller et al. have demonstrated that GP can induce chemoresistance of Gemcitabine (Gem, the first-line drug of chemotherapy for PC patients) in PC patients by metabolizing Gem into an ineffective form, providing that gut bacteria contribute to worse outcomes of PC patients by making chemotherapeutic drug invalid [4]. Furthermore, Pushalkar et al. found that gut microbes are present in murine PC models, indicating that the intestinal bacterium may be transferred into the tumor environment [5]. To confirm it firmly, Pushalkar conducted an experiment and found the translocation of Gram-negative proteobacteria into the pancreas, providing that gut bacteria can migrate to the pancreas. They also found that targeting microbes by oral antibiotics can decrease tumorigenesis in PC. Above all, we deduced that regulating the gut microbiome may become a brand new method to improve the clinical outcomes of PC. However, what is the specific PC microbe that results in the development and chemoresistance of PC? To figure it out clearly, we conducted a bioinformatic analysis to detect the mechanism of gut microbes influencing the development and chemoresistance of PC. Over the previous few decades, microarray technology and bioinformatic analysis have been extensively applied to dig out the differentially expressed genes (DEGs) and its potentially functional pathways involved in the carcinogenesis of PC. In order to reduce the false-positive rates in a single microarray analysis; thus, in the present study, 3 microarray datasets were downloaded and gradually analyzed to reach DEGs between PC tissues and noncancerous tissues from Gene Expression Omnibus (GEO). Thereafter, the subsequent analysis methods such as Kyoto Encyclopedia of Genes and Genomes pathway enrichment analysis (KEGG), protein-protein interaction network analyses (PPI), Gene Ontology (GO), and survival analysis were all utilized to let us have a better understanding of the mechanism of E. coli influencing PC progression. In this manuscript, we aimed to investigate the efficacy of potential genes to promote metastasis and progression of PC by influencing the gut or tumor microbes and to explore the underlying molecular mechanisms. To deepen our common knowledge of the carcinogenesis of gut microbes, we focused on discovering whether E. coli could reduce the survival of PC patients.

## 2. Materials and Methods

### 2.1. Bioinformatic Analysis

#### 2.1.1. Data Collection

Three gene expression datasets (GSE27890, GSE46234, and GSE107610) were downloaded from the GEO database (http://www.ncbi.nlm.nih.gov/geo) [6]. The GSE27890 dataset included 4 PC tissue samples and 4 noncancerous samples. GSE46234 contained 4 PC but 4 normal samples. GSE107610 contained 45 PC samples and 2 noncancerous samples.

#### 2.1.2. Differential Expression Analysis

Differential expression analysis was performed for each gene chip by R software (http://www.rproject.org/) with the “Limma” package. We considered logFC (fold change) > 1 and *P* < 0.01 significant statistically. The adjusted *P* values and Benjamini and Hochberg false discovery rate were used to find out a balance between false positives and discovery of statistically significant genes.

#### 2.1.3. Enrichment Analyses of DEGs

KEGG analyses and GO were utilized with DAVID (The Database for Annotation, Visualization and Integrated Discovery; http://david.ncifcrf.gov) (version 6.7) [7], with R packages (“clusterProfiler” and “Pathview”) to figure out the masked biological processes and potential pathways. Biological analyses were utilized by using the DAVID online database to clarify the function of DEGs with R packages (“Hmisc” and “ggplot2”) for clearly viewing the function by bubble plot picture. We consider *P* < 0.05 significant statistically.

#### 2.1.4. PPI Network and Identification of Related Genes

PPI network was established based on DEGs by STRING (the Search Tool for the Retrieval of Interacting Genes). A combined score > 0.4 was considered statistically significant. Visualization of the network was performed by using a software program Cytoscape (https://cytoscape.org/) [8]. To figure out PC-related genes in the PPI network, the Cytoscape plugin “cytoHubba” was used to calculate the degree. Functional interactions between proteins could give us a novel perspective of the latent mechanisms of metastasis and progression of PC. The MCODE (plug-in Molecular Complex Detection) in Cytoscape is aimed at finding the potential and significant connected regions with MCODE scores > 5, max depth = 100, node score cut − off = 0.2, *k* − score = 2, and degree cut − off = 2.

#### 2.1.5. Survival Analysis for Extracted Genes

The outcomes of the differential expression analysis were utilized and analyzed by serving GEPIA (Gene Expression Profiling Interactive Analysis; http://gepia.cancer-pku.cn/) [9] which is based on the Cancer Genome Atlas (TCGA) and the Genotype-Tissue Expression (GTEx) project with large sequencing data from 9736 tumors and 8587 normal samples. We used the GEPIA tool to analyze the survival analysis and prognostic significance of extracted genes. Moreover, we can form the disease-free survival (DFS) curve pictures as well as the overall survival (OS) curve pictures by GEPIA with the Kaplan-Meier method (we defined *P* < 0.05 as statistical significance). We use another plugin of Cytoscape Biological Networks Gene Oncology tool (BiNGO) to help us analyze the biological process of extracted genes. Hierarchical clustering of hub genes were constructed using the UCSC Cancer Genomics Browser (http://genome-cancer.ucsc.edu/) [10].

## 3. Results

### 3.1. DEGs in PC

**Figure 1:**
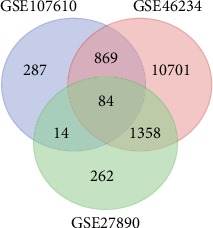
Venn diagram DEGs were selected with *P* < 0.01 and fold change > 2 among the mRNA expression profiling sets GSE27890, GSE46234, and GSE107610. A total of 84 overlapping genes were obtained.

**Figure 2:**
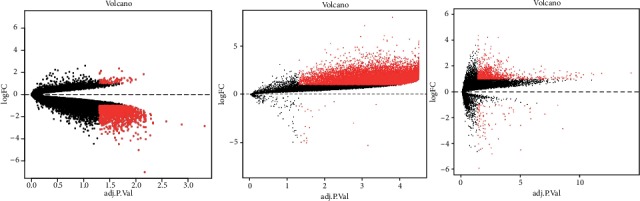
Volcano plots of differentially expressed RNAs in each gene chip. (a) GSE 27890, (b) GSE 46234, and (c) 107610 volcano plots were produced with “plots,” a built-in function of R.

A total of 13012 DEGs in GSE46234, 1718 in GSE27890, and 1254 in GSE107610 were found after standardization of the microarray results. Among the 3 datasets, 84 genes were overlapped in the datasets which can be visualized in the Venn diagram (Figure 1(a)). Moreover, we established volcano plots in order to clearly and straightforwardly view the DEGs in each gene chip. We found that there are 1254 DEGs in the GSE27890 with 79 upregulated RNAs and 1639 downregulated RNAs (Figure 2(a)). 13012 DEGs were identified in the GSE46234 gene chip. 12992 DEGs in GSE46234 were upregulated and 20 were downregulated (Figure 2(b)). A sum of 1254 DEGs were clarified in the GSE107610 gene chip, of which 1166 DEGs in GSE107610 were of high expression while 88 were of low expression (Figure 2(c)). Eventually, we combined those DEGs, respectively, from 3 gene chips and we ranked them by a robust method.

### 3.2. Enrichment Analyses of DEGs

**Figure 3:**
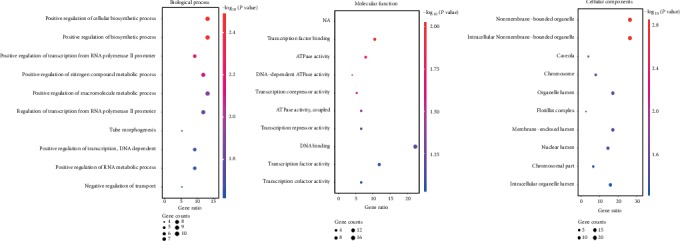
Functional and pathway enrichment analyses of the overlapping DEGs in PC. (a) BP, (b) MF, (c) CC, and (d) KEGG pathway analyses. The *x*-axis represents the *q* value (-log_10_), and the *y*-axis represents the GO term. The GO terms were measured by the rich factor, *Q* value, and number of genes enriched. The greater the rich factor is, the greater the degree of enrichment and the greater the *P* value (0, 1). The brighter the color of red is, the more significant the term.

To investigate the function, potential pathway, and biological classification of those DEGs, enrichment analyses were finished by using DAVID. Analysis results indicated that outcomes in biological processes (BP) of DEGs were significantly enriched in the regulation of the cellular biosynthetic process, biosynthetic process, transcription from RNA polymerase II promoter, and nitrogen compound metabolic process (Figure 3(a)). Outcomes in molecular function (MF) were mainly enriched in transcription factor binding (Figure 3(b)). Changes in the cell component (CC) of DEGs were mainly enriched in a nonmembrane-bounded organelle and intracellular nonmembrane-bounded organelle (Figure 3(c)). KEGG pathway analysis revealed that the upregulated DEGs were mainly enriched in pathogenic E. coli infection which provide us a theoretical basis of the relationship between pathogenic E. coli infection and progression of PC. A sum of 3 genes were testified as tumor-related genes that are associated with pathogenic E. coli infection. The full names, short names, and functions for the related genes are listed in Table 1.

**Figure 4:**
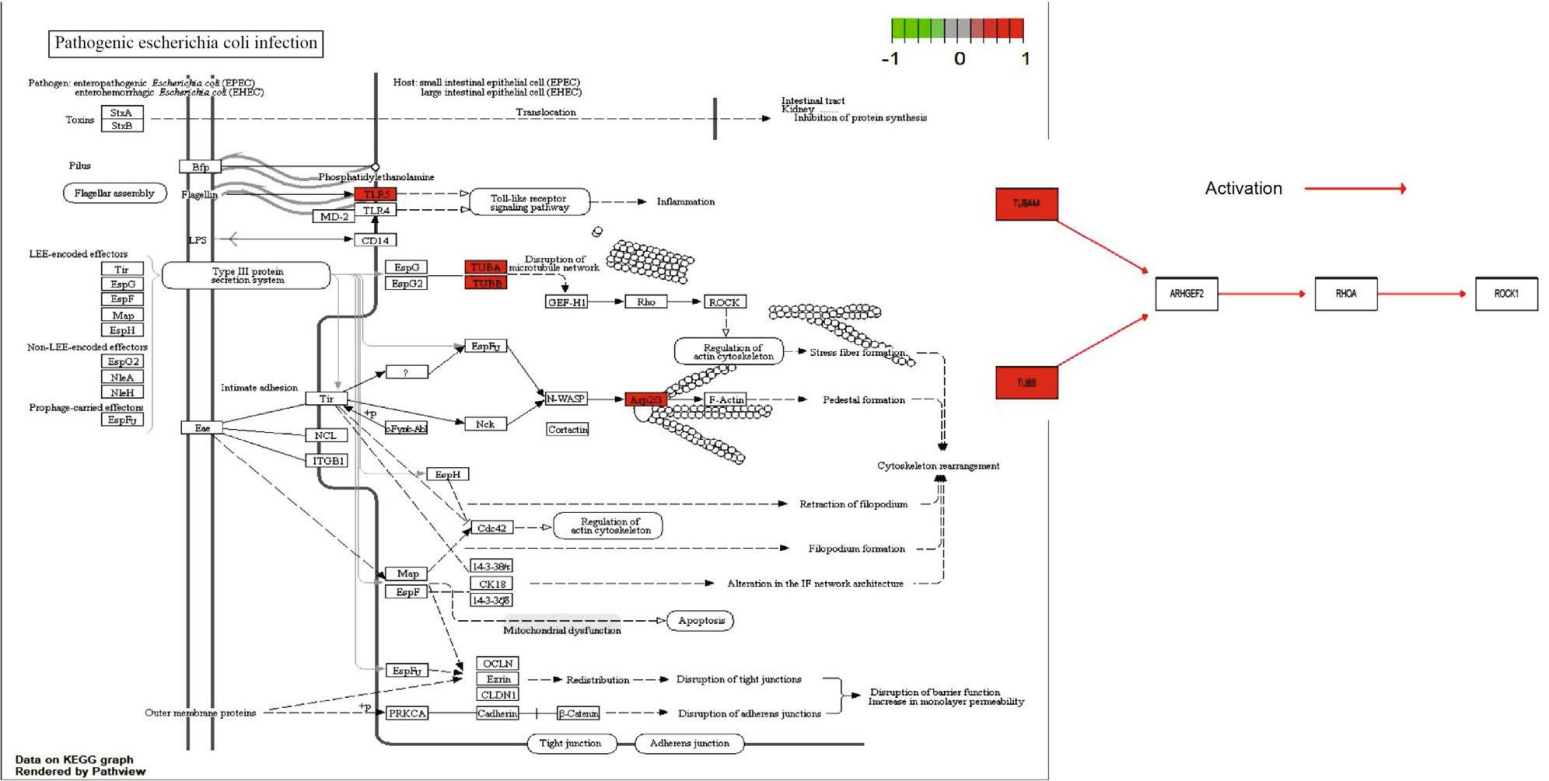
Pathway on KEGG graph by Pathview: pathogenic Escherichia coli infection and TUBB/Rho/ROCK signaling.

We subsequently used the KEGG pathway analysis and found that the TUBB/Rho/ROCK pathway plays a significant role in the progression of PC through the infection of Escherichia coli (Figure 4). Therefore, we assumed that the pathogenic E. coli infection may induce the expression of TUBB and then stimulates its downstream signaling Rho/ROCK pathway, which eventually promotes the progression of PC.

### 3.3. PPI Network Construction and Module Analysis

**Figure 5:**
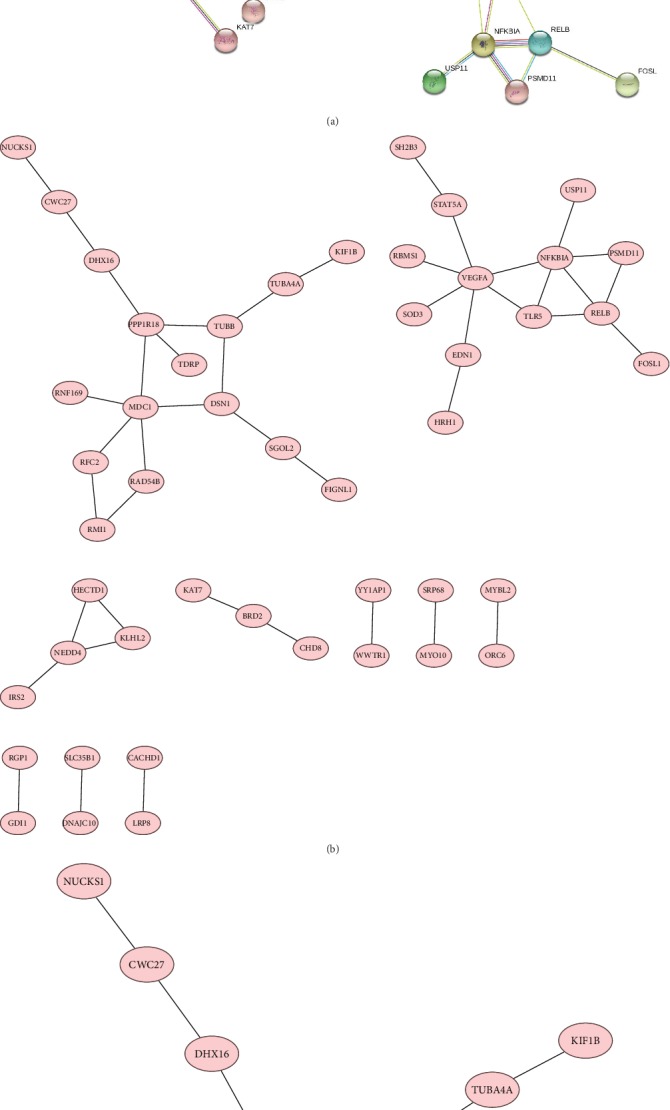
(a) PPI network analysis of the overlapping DEGs. (b) The PPI network of DEGs was constructed using Cytoscape. (c) The PPI network of DEGs was constructed using Cystoscape, and the TUBB gene has been extracted subsequently.

The PPI network of DEGs was constructed (Figure 5(a)), and the most significant module was obtained using Cytoscape (Figure 5(b)). TUBB-associated PPI network was extracted to clarify the potential targets (Figure 5(c)).

### 3.4. Overexpression of TUBB Was Found in Patients with PC

**Figure 6:**
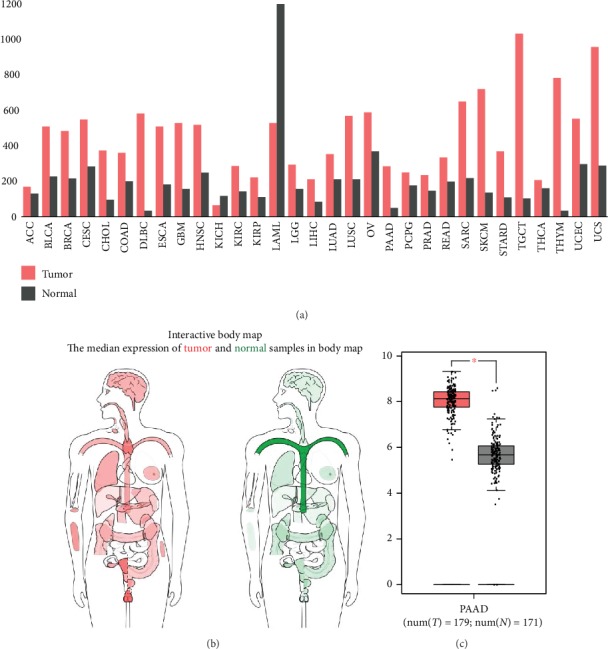
(a) Expression of the TUBB gene between all tumor samples and normal tissue (bar plot). (b) Differential expression of TUBB between PC and normal tissue in body map and (c) on box plots (N: normal; T: tumor).

We used GEPIA to extract expression data from various types of cancers in order to clarify the differential expression of TUBB in PC and other potential tumors. We found that TUBB was upregulated and overexpressed in various tumors including PC (Figure 6(a)). To further investigate the expression of TUBB in PC, we also certified that TUBB was significantly overexpressed in PC compared with normal tissues by body map and box plots (Figures 6(b) and 6(c)).

### 3.5. Overexpression of TUBB Showed Shortened Overall Survival in Patients with Pancreatic Cancer

**Figure 7:**
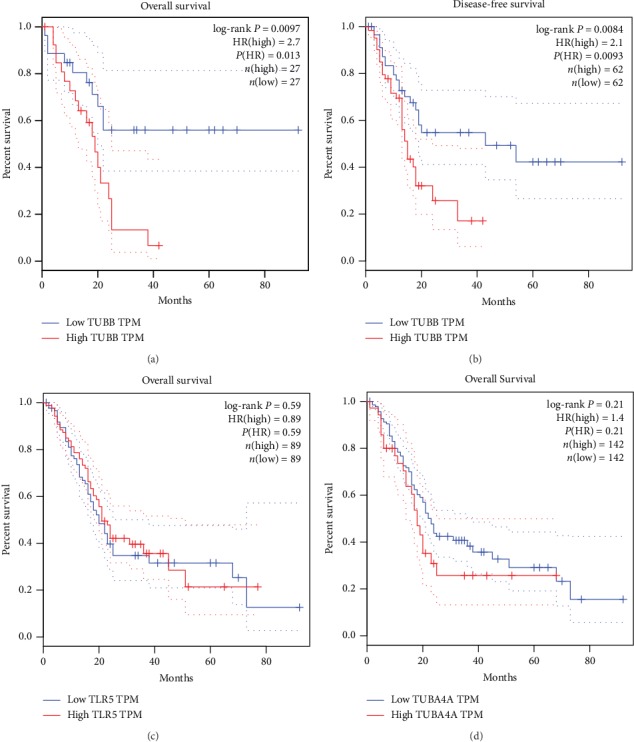
(a) Overall survival and (b) disease-free survival analyses of TUBB. Overall survival analysis of (c) TLR5 and (d) TUB4A and was performed using Gene Expression Profiling Interactive Analysis (GEPIA) database online platform. *P* < 0.05 was considered statistically significant.

Survival analysis of the related genes was analyzed using the GEPIA online platform. The overall survival analysis of the related genes was performed using Kaplan-Meier curve (Figure 7). PC patients with an overexpression of TUBB showed worse overall survival (log-rank *P* = 0.0097, HR = 2.7) than those with a low expression of TUBB. It showed no significant difference in TLR5 alterations (log-rank *P* = 0.59, HR = 0.89) and TUBA4A alterations (log-rank *P* = 0.21, HR = 1.4) (Figures 7(a), 7(c), and 7(d)). Besides, we conducted a disease-free survival analysis for PC patients between the overexpression and low expression of TUBB and found that overexpression of TUBB also showed worse disease-free survival than those with a low expression of TUBB (log-rank *P* = 0.0084, HR = 2.1) (Figure 7(b)).

## 4. Discussion

Microbes are essential compositions of the PC tumor microenvironment [11]. Gut microbes are recently regarded as vital drivers for promoting metastasis of PC and enhancing the crosstalk among immune system as well as carcinogenesis of PC. The function of gut and tumor microbes towards PC metastasis and progression is more available and effective than what we have expected. There are strong interactions between the immune system and microbes, which is important for inducing immune response to PC progression [12]. The infection of specific microbes stimulates the axis of the immune system, signaling pathway, and PC cells, affecting immune response and PC development as a result [13].

Interestingly, recently, a researcher detected the same variety of gut microbes in pancreatic microbes maybe because gut microbes could be transported by the circulatory system or the biliary-pancreatic duct to the pancreas [14]. Moreover, there are significantly different amounts and varieties of microbes between the normal pancreatic tissue and PC tissue, which means the specific microbes in a tumor may be firmly related to the development of PC [15]. Riquelme et al. collected stools from PC patients and detected the compositions. They found that human gut microbiome overlap 25% of the human tumor microbiome, while none from the normal tissue. Obviously, it indicated that the gut microbes could colonize the pancreas and influence bacterial diversity and composition in a tumor, as well as regulate immune response of PC patients [16]. Therefore, we concluded that the gut bacteria can modulate tumor bacteria composition to regulate the progression of PC and the survival rate of PC patients.

Notably, patients with overexpression of TUBB may result in low overall survival rate compared with patients with a low expression of TUBB in survival analysis. As known to us, tubulin has two heterodimers (tubulin*α* and tubulin*β*). In most tumor cells, the highly expressed isoform of tubulin*β*5 is encoded by the TUBB gene [17]. A previous study has demonstrated that mutations of TUBB are closely associated with both chemoresistance and worse overall survival in non-small-cell lung cancer [18]. Through the KEGG pathway analysis, we discovered that TUBB was correlated with the activation of the Rho/ROCK signaling pathway. Our study is the first article to discuss the influence of the gut microbes on clinical outcomes of PC by bioinformatic analysis and the first article to deduce that TUBB plays a vital role in pathogenic E. coli infection by regulating TUBB/Rho/ROCK signaling. Hence, here comes the question: What is the function of the Rho/ROCK signaling pathway?.

The stimulation of the Rho/ROCK signaling pathway is essential for cell proliferation, migration, and differentiation. Rho guanosine triphosphatases are the main regulatory proteins of actin cytoskeleton which may be closely associated with tumor metastasis [19]. Researchers have proved that the activation of Rho–ROCK family kinase pathway results in cancer metastasis [20]. Another study demonstrated that an inhibitor of Rho signaling such as 8-hydroxydeoxyguanosine can inhibit PC metastasis by suppressing the RhoA-ERM- (ezrin-radixin-moesin-) CD44-EMT (epithelial-to-mesenchymal transition) pathway [21]. Moreover, the application of the Rho inhibitor Fasudil has been reported to inhibit PC progression by impairing matrix contraction and disruption of collagen matrix integrity [22]. There is emerging evidence that Rho/ROCK signaling could lead to the progression of PC, but the definite relationship between TUBB and Rho/ROCK signaling pathway has not been well studied. This article showed that TUBB can induce the activation of Rho/ROCK signaling which clearly showed the relationship between the TUBB and Rho/ROCK signaling pathway. Moreover, we testified that the TUBB/Rho/ROCK signaling pathway is inseparably correlated with the infection of gut bacteria E. coli.

Gut bacteria have been widely explored in recent years. It has been demonstrated that diverse gut microbes can contribute to marvelously positive outcomes of PC, but imbalance of gut microbes may conduct adverse impacts [1]. In this article, we conducted KEGG pathway analysis and found that the TUBB/Rho/Rock pathway may play a significant role in the progression of PC which may be associated with E. coli.

E. coli is one kind of Gram-negative Gammaproteobacteria. A recent report demonstrated that gammaproteobacteria can produce bacterial enzyme cytidine deaminase (CDD) which can metabolize activated Gem into an inactive type (2′,2′-difluorodeoxyuridine). Gem is widely used to treat PC. Hence, inactivation of Gem metabolized by CDD that is produced by E. coli might contribute to the chemoresistance of PC. A prominent study concluded that E. coli has the ability to produce CDD and culminate in the CDD-mediated gem resistance. But this behavior can be alleviated by the antibiotic ciprofloxacin, which provide a novel therapeutic method that targets the gut bacterium which may have the potential to treat chemoresistance of PC patients [4].

Mechanically, we imagined that the gut microbes may regulate the immune system and inflammation. The outcomes of recent researches surprisingly supported it. First, microbes can recruit the proinflammatory cells and induce the cells to secrete proinflammatory cytokines, which as a result promotes tumor metastasis. Second, the inflammation induced by microbes can increase angiogenetic factors and eventually improve the living condition of cancer cells. Third, imbalance of gut microbes in the tumor can inhibit the immune system to attack the tumor by suppressing CD8+ T cells [16]. Therefore, we reckon that the mechanism of microbes contributing to the metastasis of PC may be the consequence of both inflammation activation and the innate immune system being no longer in effect. Butyrate, a short-chain fatty acid, is a metabolic production from the gut bacteria, which can combine with specific G protein-coupled receptors in immune cells, culminating in antimicrobial immune responses by activating peroxisome proliferator–activated receptor-*γ* (a nuclear receptor) in colonocytes. This finding indicates the close link and intertalk between immune cells, gut bacterium, and colonocytes [23, 24].

Pushalkar et al. put up with the idea that gut and tumor microbes can promote PC progression by inhibiting the immune system and increasing the immune checkpoint PD-1 expression on T cells [5]. The immune tolerance and immune inhibition induced by the gut and tumor microbes may be the consequence of higher pattern recognition receptor (PRR) activation in the tumor microenvironment. The microbial pattern recognition of Toll-like receptors (TLR) represents a very powerful proinflammatory stimulation and the binding of specific antigenic microbe-associated molecular pattern contributes to carcinogenesis. TLR, which is one member of PRP, plays a vital role in immune response to microbial infection. TLR can promote metastasis of PC through regulation of immune suppression and inflammation in desmoplastic stroma. Mechanically, TLR4 bind to damage-associated molecular patterns (DAMP) inducing the recruitment of myeloid differentiation factor 88 which as a result promote carcinogenesis of PC by activating the NF-*κ*B and mitogen-activated protein kinase (MAPK) signaling pathways [25]. In PC of animal models, inflammation induced by gut microbes can stimulate the activation of carcinogenic pathways, such as MAPK, KRAS, and NF-*κ*B [26].

Experimental evidence in a mouse model of PC proved that the activation of TLR4 can promote pancreatic tumor development through activating the NF-*κ*B and MAPK signaling pathways in immune cells [25]. Above all, we found that microbes leading to the progression of PC are closely correlated with immune suppression as well as proinflammatory effects and checkpoint-related immunotherapy.

Ablation of the gut microbes via antibiotics has been reported to have the ability to inhibit PC growth and metastasis by enhancing tumor-related immunity in PC through mouse model experiments [27]. Therefore, the combination of antibiotics and inhibitors of immune checkpoints as well as inhibitors of protumor signaling (TUBB/Rho/ROCK) will provide novel possibilities for treating patients with PC.

Concretely, we deduced that the gut microbiome may influence the survival of PC patients by regulating the TUBB/Rho/ROCK signaling pathway and affecting immune system response. Moreover, we proposed that gut bacteria examination may become a potential diagnostic tool to detect the progression of PC. Besides, novel drugs targeting the tumor-associated microbes in the early stage of PC patients may be a promising therapeutic method to improve the survival of PC patients. Targeting the microbes may reverse the immune tolerance of tumor and strengthen immunotherapy. Novel strategies may be adopted in co-treatment with antibiotics and inhibitors of protumor signaling (TUBB/Rho/ROCK), which may bring a brand new hope for patients with PC.

This study has some limitations that deserve comments. First, there is not enough data from the current database to support the relationships between TLR5 or TUBA4A and PC. Second, this manuscript use bioinformatic analysis to provide a novel idea and theoretical basis for the direction of future clinical trials, but it lacks the support from clinical trials and experiments in present.

Above all, we have a better understanding of the significant role of the gut and PC microbes in progression of PC.

In conclusion, we suggest that the anti-tumor drug that target the TUBB/Rho/ROCK signaling pathway may be co-treated with oral antibiotics targeting E. coli, which may activate T cells, strengthen immune protection, boost immune surveillance, and enhance sensitivity to immunotherapy in PC. The specifical clinical outcome of targeting the gut and tumor bacteria on tumor immunity needs to be further explored. However, the future of researches between microbes and PC progression will be brightly new.

## Figures and Tables

**Table 1:** Basic information on the 3 related genes.

Species	Gene title	Gene symbol	Gene Ontology molecular function
Homo sapiens	Tubulin, beta class I	TUBB	Nucleotide binding GTPase activity Structural molecule activity Traceable author statement Structural constituent of cytoskeleton GTP binding Protein domain-specific binding Protein complex binding GTPase-activating protein binding MHC class I protein binding

Homo sapiens	Tubulin, alpha 4a	TUBA4A	Nucleotide binding RNA polymerase II core promoter proximal region Sequence-specific DNA binding transcription factor activity GTPase activity Protein kinase activity Protein serine/threonine kinase activity Nonmembrane spanning protein tyrosine kinase activity Structural constituent of cytoskeleton Protein binding ATP binding GTP binding Kinase activity

Homo sapiens	Toll-like receptor 5	TLR5	Transmembrane signaling receptor activity Inferred from electronic annotation Interleukin-1 receptor binding Inferred from physical interaction Protein binding Inferred from electronic annotation

## Data Availability

The bioinformatic data used to support the findings of this study are included within the article.
